# Reaching 80 Years of Age: Clinical, Behavioral, and Psychosocial Related Risk Factors in a Large Cohort of Israeli Working Men

**DOI:** 10.3390/jcm10235706

**Published:** 2021-12-05

**Authors:** Shahar Lev-Ari, Anne Marie Novak, Adva Zemer, Yariv Gerber, Uri Goldbourt

**Affiliations:** 1 Department of Health Promotion, School of Public Health, Sackler Faculty of Medicine, Tel-Aviv University, Tel-Aviv 69978, Israel; annemarien@mail.tau.ac.il (A.M.N.); advazemer@mail.tau.ac.il (A.Z.); 2 Department of Epidemiology and Preventive Medicine, School of Public Health, Sackler Faculty of Medicine, Tel-Aviv University, Tel-Aviv 69978, Israel; yarivg@tauex.tau.ac.il (Y.G.); goldbu1@tauex.tau.ac.il (U.G.)

**Keywords:** longevity, male longevity, mid-life risk factors, family problems, psychosocial risk factors, behavioral risk factors, clinical risk factors, reaching 80, lifespan

## Abstract

The objective of this study was to estimate the probability of long-term overall survival based on total number of risk factors (RF). We also sought to examine the role of midlife clinical, behavioral, and psychosocial predictors of longevity in a large cohort of Israeli men. This study was based on the Israeli Ischemic Heart Disease (IIHD) cohort that included over 10,000 men who were followed up for mortality over more than four decades. During the 43 years of follow-up, 4634 (46.1%) men survived to 80 years of age or older. We considered cigarette smoking, diabetes mellitus, high systolic blood pressure, hypercholesterolemia, low socioeconomic status, and serious family problems as RF at ages 40–65. Cox proportional hazards regression models, with age as the time scale, were constructed to estimate the hazard ratios (HRs) for failure to survive 80 years of age. Compared with men free of all the above RF, those with one identified RF (HR = 1.58, 95% CI: 1.42–1.75) and counterparts with two identified RF (HR = 2.18, 95% CI: 1.96–2.43) were at a significantly greater risk of death before 80. Additional RF further increased the risk of early mortality (HR = 3.62, 95% CI: 1.50–8.73 for men with 5 RF). The results suggest a role of physiological, behavioral, and psychological risk factors at midlife in predicting longevity.

## 1. Introduction

The study of longevity has become one of the fastest growing and compelling fields in public health and health promotion. Questioning what leads to longer lives and better quality of life in the elderly is central to contemporary medicine. While a lot of attention has been devoted to the study of physiological health and its impact on longevity, there also exists a variety of psychosocial factors which have been associated with longer lives: positive emotions and emotional well-being [1,2], healthy familial and intimate relationships [3,4,5], and socioeconomic status (SES) [6].

Longevity has increased dramatically in the 20th and 21st centuries [6], with at least 15% of men and 20% of women born in the year 2000 being projected to reach the age of 90 [7]. While significantly more people reach advanced age, the maximum recorded lifespan remains stagnant, with very few people living beyond 100 [6]. Global life expectancy at birth is currently 72.6 years [7], 78.7 in the United States [8], and 82.8 years in Israel [9]. Men continue to die younger than women, with life expectancy at birth for Israeli men at 81.0 years, 3.7 years shorter than for women [7,9]. Despite the milestones achieved in recent years, reaching 85 years of age remains rare, and every year beyond 90 was recognized as “substantially more exceptional than the last” [6]. Improvements in the environment and in pharmacotherapy brought advancements in life expectancy, yet there are many more factors at play. In the past century, infant and maternal mortality dropped significantly, while the living and working conditions continued to improve, synergistically lengthening the life expectancy at birth. Medicine was able to halt most infectious disease, treat previously incurable illnesses, and stop the progression of many more. Environmental, psychosocial, and cultural factors that are associated with different regions of the world may affect longevity as well, beyond the genetic predispositions of the elderly populations studied [6]. Studies show that genetic predisposition and physiological health are not the only factors at play: the Georgia Centenarian Study revealed that the quality of life as well as economic and emotional well-being all impacted longevity [1]. The findings suggest that 62.5% of octogenarians and 47.5% of centenarians satisfied these three components of the proposed model of successful aging [1].

Early recognition of the modifiable physiological and psychosocial risk factors that impact our lifespans is important, as it enables early interventions [10,11], and therefore research conducted at mid-life is especially significant. Among the well-established risk factors determining longevity recognized to date are smoking habits [12,13], diabetes mellitus [14], obesity [14], cardiovascular health [15], and sedentary lifestyle [15]. While it is generally recognized that married people live longer [16,17] and the very presence of social relationships in one’s life has a positive effect on one’s health and longevity [18,19,20], few studies so far have examined the impact of quality of midlife family relationships on survival among men. Fewer studies still focused on the conjunction of physiological, behavioral, and psychological factors and their effect on longevity. More research is needed to assess the long-term effect of multifaceted risk factors at mid-life on mortality. The recognition of these factors in the middle-aged may prove important, enabling early interventions aimed at improving health, well-being, and longevity of the population. Our aim was to examine the role of midlife clinical, behavioral, and psychosocial predictors in reaching 75, 80, and 85 years of age. We estimated the probability of overall survival based on the total number of risk factors.

## 2. Materials and Methods

This study is based on the Israeli Ischemic Heart Disease (IIHD) prospective cohort. The IIHD is a longitudinal investigation of risk factors for disease in Israeli men. This cohort includes a wide range of occupations and socioeconomic levels that were present in the male working population of Israel at the time of inclusion. At the time of recruitment, subjects underwent an extensive appraisal of health and behavioral patterns, including a structured psychosocial questionnaire, and were then followed up over a period of 43 years to record mortality.

The original cohort included 10,059 individuals, who were recruited by stratified sampling of civil servants and municipal employees in 1963 [21]. Participants were chosen by stratified sampling based on an age of 40 years and above on inclusion, with the place of work confined to the three largest urban areas in Israel (Tel-Aviv, Jerusalem, and Haifa). The sample was designed to include members originating from six geographical regions (central Europe, eastern Europe, the Balkans, the Middle East, and Northern Africa), in a combination approximately proportional to the general Israeli male population of a similar age at that time. 

Participants underwent clinical and blood biochemical evaluations, as previously described [20,22]. Blood pressure was measured in the right arm, with the subject in the recumbent position, 30–45 min after arrival at the clinic, and again, 15–30 min later. The procedure followed the World Health Organization guidance (Tech. Rep. Ser. 1962, vol. 231, p. 5). Diabetes mellitus was defined on the basis of anti-diabetic oral therapy or insulin use at baseline in addition to casual blood glucose levels. A two-hour glucose tolerance test was then carried out to determine the participant’s diabetic status. Calculation of body mass index (BMI) was based on height without shoes measured to the nearest centimeter, and weight to the nearest kilogram. Normal BMI was defined as raging between 18.50 and 24.99 kg/m^2^, overweight BMI as 25.00 and 29.99 kg/m^2^, and obese as 30.00 kg/m^2^ or higher. Cigarette smoking was recorded as five separate categories including never smoked, smoking cigar or pipe, quitters, and three different categories according to the amount smoked. Subjects were also classified as having ever smoked or not.

SES was assessed by categorizing education and occupation data into five strata, with the lowest stratum including laborers with an up to elementary school education, and the highest stratum including men with at least some university or equivalent education, engaged in professional or teaching work.

Subjects underwent a structured psychosocial questionnaire at baseline, based on the previous experience and pre-test of a more detailed questionnaire among port workers [23]. Trained interviewers administered the questionnaire following a detailed protocol. The aim of the questionnaire was to assess (a) the person’s attitudes to problems in certain areas in his past and present life situation; (b) his reaction to the above problems; and (c) problems relating to his past and present work, and financial and family life situation. Participants were asked whether they had experienced or were experiencing family difficulties. The questionnaire included questions such as: Do/did you currently/previously have family difficulties? Do your wife and children pay attention to you? Does family not paying attention affect you? Does your wife show her love to you? When conflicted with your wife—do you show it or keep to yourself? When hurt by your wife or children—do you tend to retaliate?

The construct validity of the questionnaire was assessed, and these parameters have previously been described in detail [20,22,23]. The answers were scored on a Likert scale (0 for no problems to 4 for 4 or more problems). Severe and very severe family problems, which were defined a priori, were scored on the family difficulties scale (i.e., serious—3 problems, very serious—4 or more problems). The outcome was recorded as a binary outcome, and the results were assessed using a multivariable model. Subjects who were never married, divorced, or widowers (~2%) were not asked to take part in this questionnaire, and were not included in further analysis related to this questionnaire.

The underlying cause of death was documented as a case-by-case determination by a review panel through the mid-1970s, and by using the International Classification of Diseases (ICD) codes thereafter. Deaths from stroke were based on ICD-9 codes 431–438, and those from CHD by codes 410, 411, 414, and 798. For the earlier (pre-1971) deaths, a comparison of death certificates with the analyses of hospital records by the panel, yielded a 90% agreement. Information on mortality from 1970 until the end of 2006 was derived from the Israeli Mortality Registry.

Patient characteristics across the baseline are presented as means and standard deviations for continuous variables, and as frequencies for categorical variables; differences between groups were examined using analysis of variance or the χ^2^ test, as appropriate. To calculate the multivariable adjusted odds ratios (ORs) for the association between physiological, lifestyle-related and psychological factors and mortality, defined as reaching 75, 80, and 85 years of age, ORs were calculated using three models: (1) the initial adjustment was made for age, diabetes mellitus, systolic blood pressure, smoking, and BMI (model 1); (2) followed by a model additionally adjusting for serious or very serious family problems; and (3) further adjusted for SES quintiles.

The central purpose of the analysis was to estimate the risk associated with none, one, two, three, four, and five or more mortality risk factors out of a total of seven risk factors. The range of these factors are categorized as following: hypertension (systolic pressure of 140 or more), hypercholesterolemia (total cholesterol above 240), diabetes, current smoking, BMI (above 30 kg/m^2^), serious family problems (3 or above), and low SES.

Cox proportional hazards regression models [24] with age as the time scale were constructed to estimate the hazard ratios (HRs), and 95% confidence intervals (CIs) for not reaching 75, 80, and 85 years of age associated with the number of risk factors (0 as referent). Using age as the time scale, subjects entered the risk set at the age they were recruited to the study (1963 or 1964) and exited at their event/censoring age. In an attempt to adjust for calendar effects, we stratified the model by birth year intervals [25]. Specifically, we used the intervals: ≤1910; 1911–1918; and ≥1919 which divide the sample into approximate tertiles. Statistical analysis was carried out using the Stata statistical software package, version 17 (Stata, College Station, TX, USA) and SAS software, version 9.4 (SAS institute, Cary, NC, USA).

## 3. Results

A total of 10,059 men were included in the analysis. Of these men, 5417 (53.9%) died before reaching 80 years of age, and were classified as non-survivors. The average age of the population at recruitment (1963) was 49.3 ± 6.9 years. Baseline characteristics across categories of survivors and non-survivors are presented in Table 1. Octogenarians had lower levels of systolic blood pressure, total cholesterol, and rates of diabetes. An assessment of psychosocial variables revealed that those surviving to 80 had fewer family problems and a higher SES. Approximately two thirds of the participants had smoked at some time, with lower rates among the survivors.

Associations of risk factors and overall survival are shown in Table 2. High (140–159 mmHg) and very high (≥160 mmHg) systolic blood pressure was associated with a reduced OR of survival to age 80 (OR = 0.56, 95% CI: 0.50–0.63) and (OR = 0.27, CI: 0.23–0.31), respectively. Elevated serum cholesterol (>240 mg/dl, OR = 0.74, 95% CI: 0.66–0.83) and a diagnosis of diabetes (OR = 0.34, 95% CI: 0.27–0.43) were associated with comparable reduction of OR. Medium and heavy smokers had lower odds of survival to reach the age of 80 (OR = 0.51, 95% CI: 0.45–0.58; OR = 0.36, 95% 0.32–40, respectively). Reported family problems (Table 2, model 2; model 1 further adjusted for family problems) was associated with a lower OR (OR = 0.42, 95% CI: 0.22–0.78). The two lowest SES strata (Table 2, model 3; model 2 further adjusted for family problems) were associated with reduced OR (OR = 0.67, 95% CI: 0.56–0.79) and 0.54 (95% CI: 0.46–0.64), respectively.

Table 3 presents the ORs of variables assessed as predictors for reaching 75, 80, and 85 years of age. Middle-aged men diagnosed with diabetes, hypertension, and hypercholesterolemia had significantly lower odds of reaching 75, 80, and 85 years of age. Serious family problems (4 or above) were associated with survival at all ages tested, particularly with the odds of reaching 75 years of age (OR = 0.29, 95% CI: 0.16–0.53). Men categorized in the two lowest socioeconomic strata (average low and low) had lower ORs for survival to ages 75, 80, and 85.

**Figure 1:**
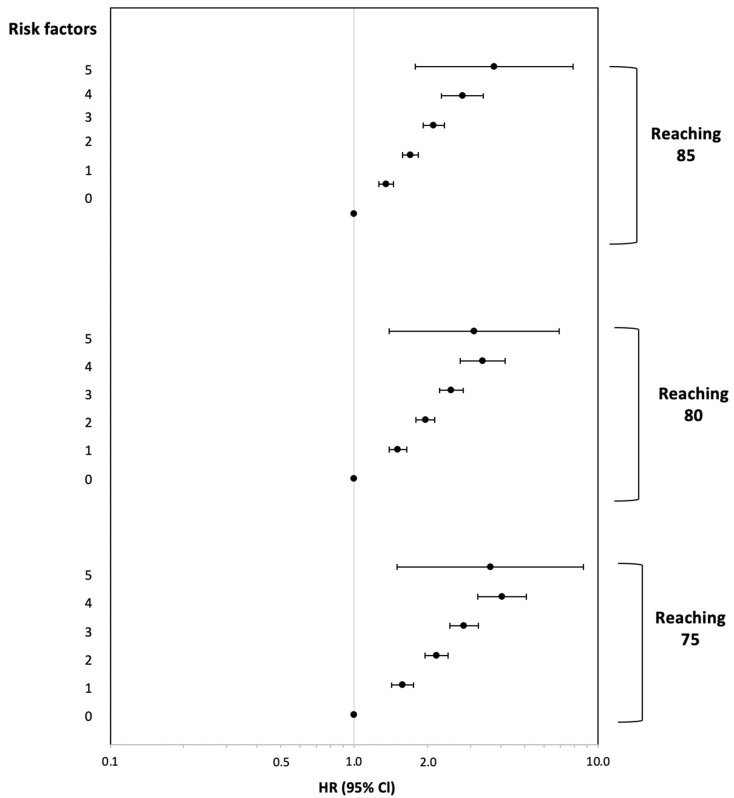
Hazard ratios and 95% confidence intervals for not reaching 75, 80, and 85 years of age associated with the number of risk factors (0 as referent). Results are presented on a logarithmic scale. Error bars indicate likelihood-ratio-based on 95% confidence intervals.

Cox proportional hazards regression models, with age as the time scale, were constructed to estimate the hazard ratios (HRs) for failure to survive 75, 80, and 85 years of age. A decline in the probability of survival, as the number of risk factors increases, was found for all ages (Figure 1). We considered cigarette smoking, diabetes mellitus, high systolic blood pressure, hypercholesterolemia, low socioeconomic status, and serious family problems as risk factors (RF) at ages 40–65. Compared with men free of all the above RF, those with one identified RF (HR = 1.58, 95% CI: 1.42–1.75) and counterparts with two identified RF (HR = 2.18, 95% CI: 1.96–2.43) were at a significantly greater risk of death before 80. Additional RF further uncreased risk of early mortality (HR = 3.62, 95% CI: 1.50–8.73 for men with 5 RF).

## 4. Discussion

In our study, we analyzed data collected on a cohort of 10,000 working middle-aged Israeli men recruited in 1963 who followed up for 43 years. The subjects represent the male working population of Israel from a range of socioeconomic backgrounds and occupations. We evaluated the effect of clinical, behavioral, and psychosocial factors at mid-life on longevity. We found that several risk factors present at mid-life were strong and independent predictors of survival past the ages of 75, 80, and 85: smoking, diabetes, high systolic blood pressure, hypercholesterolemia, low socioeconomic status, and serious family problems. Our results support previous studies that have reported the association of various risk factors in midlife with life expectancy [10,11,12,15,26,27]. A unique feature of this study was the inclusion of psychosocial factors in conjunction with biological factors in the assessment of reaching old age. Cox proportional hazards regression model, with age as the time scale, suggests a hazard ratio of 1.58 with a single risk factor, and as high as 3.62 with five risk factors.

Findings in this study may suggest a role of family problems in predicting survival at old age. Looking at rates of survival among working men reporting different degrees of serious or very serious family problems, it might appear of value to consider possible associations with longevity. These findings are supported by several analyses performed in the past on this cohort, which proved an association between psychosocial factors and disease: angina pectoris [20], stroke [23], and duodenal ulcers [22]. Stressful social relations, including frequent worries or demands in familial relationships, increased mortality among the middle aged, as seen in a large cohort from Denmark [4]. A multivariate analysis from Finland, including background physiological and sociodemographic factors as well as modifiable behavioral risk factors, confirmed the impact of stress on longevity [17]. That large prospective cohort study examined the impact of family problems, revealing a similar to the seen in our results correlation between perceived difficulties with one’s children and longevity [17]. A cohort study that examined the effect of familial relationships on mortality over 20 years found that low levels of support combined with perceived negativity from spouse increased the participants’ mortality [5]. Unsatisfactory relationships have been linked to decreased longevity [27], cognitive function [28], and cardiovascular health [20] in men. Autonomy, social support, and control are some of the psychosocial factors identified as related to longevity and healthy aging [29]. 

We found that the SES of the participants significantly affected their odds of reaching 75, 80, and 85 years of age. Men categorized in the two lowest socioeconomic quintiles (average low and low) were at a disadvantage compared to the wealthier subjects. This finding confirms what has been shown internationally in various populations [6,30,31,32]. Education is strongly connected with income, and several studies note an association between low income and mortality, which was recognized after controlling for behavioral risks and demographic factors [31]. In a study on 3617 American adults, income remained a strong predictor of 19-year mortality after controlling for health behaviors [32]. Higher mortality was also reported in unemployed and lower-employed men, with the effect lasting through their retirement age [33]. A 2008 study that followed British civil servants over 17 years to assess successful aging observed that one’s position in the social hierarchy in midlife is strongly associated with the chances of entering early old age free from major disease and with good functioning [34]. Our study extends these results in a similar cohort which was followed up for up to 45 years.

The other risk factors studied included: blood pressure, BMI, smoking, cholesterol levels, and diabetes. We found that middle-aged men diagnosed with diabetes, hypertension (systolic pressure of 130 or more), or hypercholesterolemia (total cholesterol above 200) had significantly lower odds of reaching 75, 80, and 85 years of age. These findings are consistent with some of the most important studies to date on healthy aging, including the Framingham Heart Study [14] and the Honolulu-Asia Aging Study [11]. It is important to note that, similarly to our results, not all studies conducted to date support the conclusion that lower body weight is associated with longer lifespans; a 2018 study found that an overweight or obese status was not associated with a higher mortality risk [35]. This corresponds with previous research which found that obesity in older populations may afford some protection against health shocks or conditions that lead to frailty and wasting [17,36]. We found an association between smoking and longevity, with the lowest odds of reaching the age of 80 observed in the heaviest smokers—participants who smoked 20 or more cigarettes a day were at a three times higher risk of death before reaching 80. We found a small difference between those smoking less than 10 cigarettes a day and those who quit smoking; while both groups had decreased odds of reaching 80, those who quit smoking were at a slight disadvantage compared to light smokers. The association between smoking and premature death is well-established [12,13], including in people who have quit smoking [37,38]. People who have quit smoking are likely to have done so due to poor health [39], which might explain the slight difference in longevity odds in favor of light smokers, observed in the current study. Yet, it is important to note that even very light smoking carries significant risks [40].

Cox proportional hazards regression models, with age as the time scale, were constructed to estimate the hazard ratios (HRs) for failure to survive to 75, 80, 85 years of age. A decline in the probability of survival, as the number of risk factors increases, was found for the above ages. Men with one RF (HR = 1.58, 95% CI: 1.42–1.75) and counterparts with two RFs (HR = 2.18, 95% CI: 1.96–2.43) were at a significantly greater risk of death before 80. Each additional RF increased the risk of early mortality, with men experiencing five RFs at mid-life at a 3.75 times greater risk of death before the age of 85 than men with no identified RFs at that same point in time. The Framingham Heart Study found an association between a conjunction of cardiovascular risk factors, glucose balance, smoking, and educational achievement and survival, revealing that survival until 85 years of age decreased with an increasing number of these factors [14]. Participants with only 2% of those with all five risk factors surviving until 85 [14]. Similar findings were reported in Japanese American men studied in the Honolulu-Asia Aging Study [11,19]: the probability of survival until 85 was at 9% in men with six or more risk factors, which included hypertension, hyperglycemia, body weight, alcohol consumption, and smoking [11]. The authors concluded that the best approach to lengthening the lifespan, as well as maintaining good health in old age, would be a multi-targeted approach that includes avoidance of smoking and hypertension and maintaining or enhancing insulin sensitivity and lean body mass [11]. Good cardiovascular health and avoidance of smoking are seen as especially important for longevity, with significantly greater survival in non-smokers with lower total cholesterol and blood pressure [6]. While confirming these results, our study proposes that addressing psychosocial in addition to these clinical and behavioral well-established risk factors could strengthen the multi-targeted approach aimed at increasing the human lifespan.

Our study has several limitations. We assessed risk factors at one time-point. This design does not enable control for changes in the participants’ health or psychosocial status. Repeated assessments might provide a stronger ground for analyzing these associations. Furthermore, the existence of family problems may be correlated with other—unmeasured in this study—psychosocial characteristics including depression [41] and general stress [42], as well as alcohol and substance abuse [43]. Third, only male participants were recruited in this study. Further studies are warranted to estimate both genders.

The importance of the current study lies in the recognition of predictors for longevity at mid-life—including the factors that go beyond physical health. If recognized early, the risk factors may still be modifiable and increase one’s chances of reaching old age in good health. We extend the research on cumulative known physiological and behavioral risk factors in a large cohort of Israeli men followed for approximately half a century. An advantage of the current study is the incorporation of Cox proportional hazards regression model, with age as the time scale, that permits analysis of the association between number of risk factors and survival—taking into account the recruitment age, as well as their event/censoring age. In conclusion, the results of this long-term follow-up study of approximately 10,000 tenured working males suggest the role of physiological, behavioral, and psychological risk factors at midlife in predicting longevity.

## Figures and Tables

**Table 1:** Reaching 80 years of age—characteristics of the study population.

Characteristic	*n*	Alive at 80 *n* = 4642 (46.1%)	Deceased *n* = 5417 (53.9%)	*p* Value
**Systolic Pressure ^a^ (mmHg) (%)**				*p* < 0.001
≤120	2715	1221 (45.0)	1494 (55.0)	
121–129	1511	752 (49.8)	759 (50.2)	
130–139	2189	1048 (47.9)	1141 (52.1)	
140–159	2350	984 (41.9)	1366 (58.1)	
≥160	1286	349 (27.1)	937 (72.9)	
**Total Cholesterol ^b^ (mg/dL) (%)**				*p* < 0.001
<200	4262	2125 (49.9)	2137 (50.1)	
200–239	3531	1622 (45.9)	1909 (54.1)	
>240	2101	823 (39.2)	1278 (60.8)	
**Diabetes ^a^ (%)**				*p* < 0.001
No	9572	4528 (47.3)	5044 (52.7)	
Yes	479	106 (22.1)	373 (77.9)	
**Smoking ^c^ (%)**				*p* < 0.001
Never smoked	3154	1724 (54.7)	1430 (45.3)	
Quit smoking	1676	825 (49.2)	851 (50.8)	
1–10 cigarettes p/day	1496	736 (49.2)	760 (50.8)	
11–20 cigarettes p/day	1634	661 (40.5)	973 (59.5)	
20+ cigarettes p/day	1943	621 (32.0)	1322 (58.0)	
**BMI ^d^ (%)**				*p* < 0.001
<20	449	195 (43.4)	254 (56.6)	
20–25	3773	1824 (48.3)	1949 (51.7)	
25–30	4859	2265 (46.6)	2594 (53.4)	
>30	945	342 (36.2)	603 (53.8)	
**Family Problems ^e^ (%)**				*p* = 0.008
0	5292	2529 (47.8)	2763 (52.2)	
1	2494	1143 (45.8)	1351 (54.2)	
2	1176	512 (43.0)	664 (57.0)	
3	308	134 (43.5)	174 (56.5)	
4	55	16 (29.1)	39 (70.9)	
**SES ^f^ strata (%)**				*p* < 0.001
High	2424	1421 (58.6)	1003 (41.4)	
Average high	2379	1340 (56.3)	1039 (43.7)	
Average	3141	1529 (51.3)	1612 (46.7)	
Average low	1048	507 (51.6)	541 (48.4)	
Low	995	525 (47.2)	470 (52.8)	

^a^ *n* = 10,051, ^b^ *n* = 9894, ^c^ *n* = 9903, ^d^ *n* = 10,026, ^e^ *n* = 9325, ^f^ *n* = 9987.

**Table 2:** Multivariable odds ratios (OR) and confidence intervals (CI) for predictors of reaching 80 years of age.

	Model 1		Model 2		Model 3	
**Variable**	**OR**	**95% CI**	**OR**	**95% CI**	**OR**	**95% CI**
Age (y) * 5 Years	1.07	1.04–1.11	1.09	1.05–1.13	1.09	1.05–1.12
**Systolic Pressure (mmHg)**						
≤120						
121–129	0.79	0.69–0.90	0.80	0.70–0.92	0.80	0.70–0.92
130–139	0.72	0.64–0.82	0.71	0.63–0.81	0.72	0.63–0.82
140–159	0.56	0.50–0.63	0.56	0.49–0.63	0.57	0.50–0.65
≥160	0.27	0.23–0.31	0.27	0.23–0.32	0.27	0.23–0.33
**Total cholesterol (mg/dL)**						
<200						
200–239	0.91	0.83–1.00	0.90	0.81–0.99	0.87	0.78–0.96
>240	0.74	0.66–0.83	0.72	0.64–0.81	0.71	0.63–0.80
**Diabetes (y/n)**	0.34	0.27–0.43	0.33	0.25–0.42	0.32	0.25–0.41
**Smoking**						
Never smoked						
Quit smoking	0.70	0.49–0.99	0.82	0.71–0.94	0.63	0.44–0.91
1–10 cigarettes p/day	0.79	0.70–0.90	0.81	0.70–0.93	0.81	0.70–0.93
11–20 cigarettes p/day	0.51	0.45–0.58	0.53	0.46–0.61	0.55	0.48–0.62
20+ cigarettes p/day	0.36	0.32–0.40	0.37	0.32–0.42	0.37	0.32–0.42
**BMI**						
<20						
20–25	1.19	0.97–1.47	1.09	0.87–1.38	1.08	0.87–1.36
25–30	1.17	0.95–1.44	1.10	0.87–1.39	1.08	0.86–1.35
>30	0.86	0.67–1.10	0.81	0.62–1.06	0.80	0.61–1.03
**Family problems**						
0						
1			0.93	0.84–1.03	0.93	0.84–1.03
2			0.87	0.75–0.99	0.87	0.75–0.99
3			0.88	0.69–1.13	0.88	0.69–1.13
4 or more			0.42	0.22–0.78	0.42	0.22–0.78
**SES strata**						
High						
Average high					0.79	0.66–0.96
Average					0.75	0.64–0.88
Average low					0.67	0.56–0.79
Low					0.54	0.46–0.64

* five-year age groups, OR = Odds ratio. CI = confidence interval. Model 1: age, diabetes mellitus, systolic blood pressure, smoking, BMI. Model 2: Model 1 further adjusted to serious or very serious family problems. Model 3: Model 2, further adjusted for Neighborhood socioeconomic status strata.

**Table 3:** Multivariable odds ratios (OR) and confidence intervals (CI) for predictors of 75, 80, and 85 years of age.

	Reaching 75		Reaching 80		Reaching 85	
**Variable**	**OR**	**95% CI**	**OR**	**95% CI**	**OR**	**95% CI**
Age (y) * 5 Years	1.16	1.12–1.20	1.09	1.05–1.12	1.02	0.99–1.06
**Systolic Pressure (mmHg)**						
≤120						
121–129	0.91	0.78–1.06	0.80	0.70–0.92	0.85	0.73–1.00
130–139	0.75	0.66–0.86	0.72	0.63–0.82	0.70	0.60–0.81
140–159	0.59	0.51–0.67	0.57	0.50–0.65	0.57	0.49–0.67
≥160	0.28	0.23–0.34	0.27	0.23–0.33	0.24	0.19–0.29
**Total cholesterol (mg/dL)**						
<200						
200–239	0.89	0.80–0.99	0.87	0.78–0.96	0.84	0.74–0.94
>240	0.67	0.58–0.75	0.71	0.63–0.80	0.69	0.60–0.80
**Diabetes (y/n)**	0.33	0.27–0.41	0.32	0.25–0.41	0.32	0.23–0.45
**Smoking**						
Never smoked						
Quit smoking	0.83	0.72–0.95	0.81	0.71–0.92	0.72	0.62–0.83
1–10 cigarettes p/day	0.80	0.69–0.93	0.81	0.70–0.93	0.77	0.66–0.90
11–20 cigarettes p/day	0.56	0.48–0.64	0.55	0.48–0.62	0.49	0.42–0.58
20+ cigarettes p/day	0.39	0.34–0.45	0.37	0.32–0.42	0.32	0.27–0.38
**BMI**						
<20						
20–25	1.16	0.92–1.46	1.08	0.87–1.36	1.17	0.88–1.54
25–30	1.16	0.92–1.46	1.08	0.86–1.35	1.10	0.83–1.45
>30	0.80	0.62–1.05	0.80	0.61–1.03	0.79	0.57–1.10
**Family problems**						
0						
1	0.95	0.85–1.05	0.93	0.84–1.03	0.90	0.80–1.02
2	0.89	0.77–1.02	0.87	0.75–0.99	0.72	0.60–0.85
3	0.78	0.61–1.01	0.88	0.69–1.13	0.96	0.72–1.29
4 or more	0.29	0.16–0.53	0.42	0.22–0.78	0.45	0.20–0.97
**SES strata**						
High						
Average high	0.77	0.62–0.96	0.86	0.71–1.05	0.79	0.66–0.96
Average	0.89	0.74–1.07	0.91	0.75–1.06	0.75	0.64–0.88
Average low	0.70	0.58–0.85	0.77	0.65–0.91	0.67	0.56–0.79
Low	0.66	0.54–0.80	0.71	0.60–0.84	0.54	0.46–0.64

* Five-year age groups, OR = Odds ratio. CI = confidence interval.

## Data Availability

Data might be available upon direct communication with Prof. Goldbourt, an author in this publication, and the PI for the IIHD study database.
